# Exploring the clinical transition of engineered exosomes designed for intracellular delivery of therapeutic proteins

**DOI:** 10.1093/stcltm/szae027

**Published:** 2024-06-05

**Authors:** Minseong Kim, Hojun Choi, Deok-Jin Jang, Hye-Jung Kim, Yujin Sub, Heon Yung Gee, Chulhee Choi

**Affiliations:** ILIAS Biologics Inc., Daejeon 34014, Korea; ILIAS Biologics Inc., Daejeon 34014, Korea; ILIAS Biologics Inc., Daejeon 34014, Korea; Department of Ecological Science, College of Ecology and Environment, Kyungpook National University, Sangju 37224, Korea; ILIAS Biologics Inc., Daejeon 34014, Korea; Department of Pharmacology, Graduate School of Medical Science, Brain Korea 21 Project, Yonsei University College of Medicine, Seoul 03722, Republic of Korea; Department of Pharmacology, Graduate School of Medical Science, Brain Korea 21 Project, Yonsei University College of Medicine, Seoul 03722, Republic of Korea; ILIAS Biologics Inc., Daejeon 34014, Korea

**Keywords:** extracellular vesicles, exosomes, protein therapeutics, exosome purification, inflammation, drug delivery system

## Abstract

Extracellular vesicles, particularly exosomes, have emerged as promising drug delivery systems owing to their unique advantages, such as biocompatibility, immune tolerability, and target specificity. Various engineering strategies have been implemented to harness these innate qualities, with a focus on enhancing the pharmacokinetic and pharmacodynamic properties of exosomes via payload loading and surface engineering for active targeting. This concise review outlines the challenges in the development of exosomes as drug carriers and offers insights into strategies for their effective clinical translation. We also highlight preclinical studies that have successfully employed anti-inflammatory exosomes and suggest future directions for exosome therapeutics. These advancements underscore the potential for integrating exosome-based therapies into clinical practice, heralding promise for future medical interventions.

## Graphical Abstract

**Graphical Abstract:**
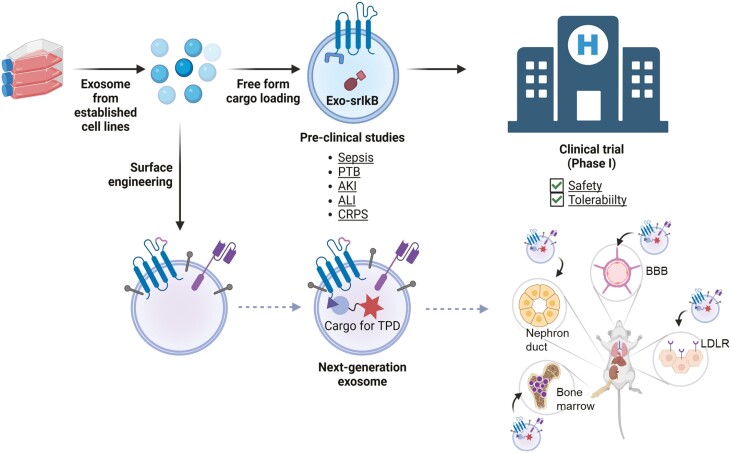

**Significance statement**

Engineered exosomes show great potential as novel drug delivery systems and therapeutic agents. By engineering payload incorporation and surface modification, they facilitate intracellular delivery of therapeutic drugs with excellent biocompatibility and safety profiles. Although clinical advancement of engineered exosomes is currently in its infancy, preclinical studies involving anti-inflammatory exosomes have provided strong evidence supporting their capacity for precisely targeted delivery and effective target engagement.

## Introduction

Drug delivery systems (DDSs) play a pivotal role in minimizing the off- and on-target side effects of therapeutics by optimizing their pharmacokinetic and pharmacodynamic properties.^1^ The development of heterobifunctional compounds, including proteolysis-targeting chimeras, has recently expanded the realm of druggable targets to include undruggable targets.^2^ These new modalities exhibit greater potency and efficacy than conventional small-molecule inhibitors, suggesting that inaccurate targeting of unintended tissues or organs could lead to pronounced detrimental effects.

Extracellular vesicles (EVs) are small, membrane-bound structures composed of lipids, nucleic acids, and proteins.^3^ They are classified into 3 main categories based on size and biogenesis: exosomes (30–200 nm), microvesicles (MVs) (100–1000 nm), and apoptotic bodies (> 1000 nm). Notably, EVs can pass through biological barriers, including the placental and blood-brain barrier (BBB).^4,5^ They are also considered safe partly because of their immune tolerance. A diverse range of active pharmacological ingredients (APIs), such as chemical-based small molecules, biologics, and nucleic acid-based drugs, can be loaded into EVs using various methods. EVs can improve the stability of APIs by protecting the encapsulated drugs from enzymatic degradation and antibody-mediated clearance. Furthermore, the innate ability of EVs to be delivered to specific tissues or cell types can be enhanced by additional surface engineering with targeting moieties, such as peptides and antibodies.^6^ Despite challenges, such as scalable production and quality control, these advantages make EVs attractive and promising drug carriers. Among subsets of EVs, we will focus on the exosomes and its application to therapeutics.

In this review, we highlight the emerging strategies of exosome engineering, with particular emphasis on the incorporation of protein payloads and surface engineering techniques for targeted delivery. Here, we present findings from a preclinical study that successfully utilized engineered exosomes to treat inflammatory diseases. Furthermore, we discuss the future directions and perspectives of engineered exosomes in terms of on-target delivery strategies and the incorporation of biological heterobifunctional degraders as a next-generation therapeutic load.

## Engineering exosomes as next-generation therapeutics

Recently, naïve exosomes derived from various types of human stem cells have been widely tested in various human diseases as it is regarded as a safer alternative to stem cell therapy. Compared to stem cells, stem cell-derived exosomes inherit the therapeutic potential of its originated stem cells while having no ethical issues, low risks of tumorigenesis, and immune response.^7^ Particularly, exosomes secreted by bone marrow derived mesenchymal stromal/stem cells (BM-MSCs) have demonstrated tissue repair and immunomodulatory functions in several diseases, including multiple signaling regulation by miRNA or long non-coding RNAs (lncRNAs) in bone fracture healing,^8-11^ chondrocyte protection in osteoarthritis,^12^ and promotion to M2 macrophages in spinal cord injury.^13,14^ However, it still has limitations such as low productivity, heterogenous nature, and so forth. Moreover, heterogenous expression of procoagulant tissue factor CD142 among MSCs is pointed out as a risk factor for blood clotting.^15,16^ Since exosomes share the main features with source cells, there would be a potential risk of thrombosis or thromboembolism when using MSCs-derived exosomes. These limitations could be overcome with the development of engineered exosomes that can knock out or modify risk factors from producing cells. In addition to this, a growing body of evidence indicates that the pharmacodynamic and pharmacokinetic properties of therapeutic exosomes can be substantially improved by incorporating a single API as a payload and by conducting surface modifications for targeted delivery.^17-20^

Engineered exosomes can be defined as exosomes with artificial modifications that overcome the limitations of naïve exosomes. In most cases, engineered exosomes are produced by established cell lines to ameliorate heterogeneity issues associated with naïve exosomes. The main advantage of engineered exosomes from established cell lines is the notable increase in productivity; large-scale cultures, expanding up to hundreds of liters, are possible with immortalized suspension cell lines, thereby improving the productivity of exosomes. As established cell lines are almost homogeneous and engineered exosomes are produced from single cell-derived, stably transfected cells, the chemistry, manufacturing, and control (CMC) aspects have become more streamlined. Moreover, the use of a single API enables the precise determination of the therapeutic mechanism of action. In addition, the adaptability of the production process allows for the development of a variety of surface-engineered exosomes targeting various diseases while using the same culture media and methodologies.

**Figure 1.**
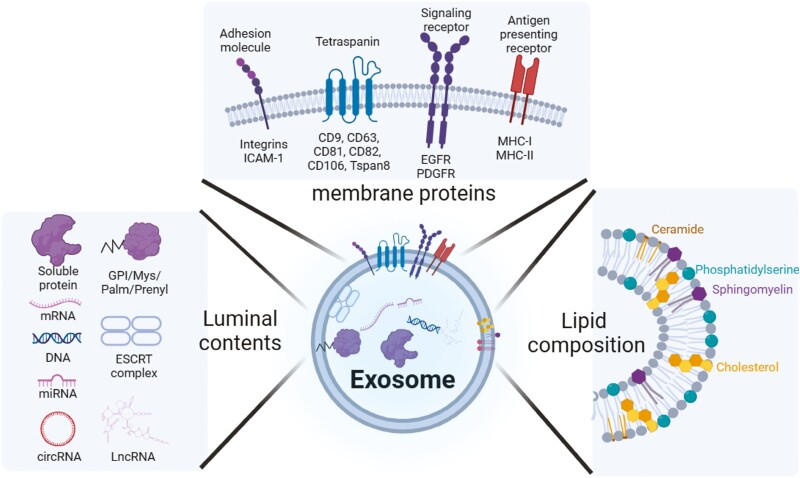
Exosome constituents. Exosomes originate from endocytosis of lipid raft, resulting in a lipid composition similar to lipid raft. Local concentrations of cholesterol and sphingomyelin enable curvature of exosomal membrane, whereas phosphatidylserine located in the outer leaflet of the membrane creates negative charge of exosomes. Inverse cornical 3D conformation of tetraspanin also contributes to maintaining the curvature of exosomal membrane. In addition, adhesion molecules, as well as signaling receptors, confer exosomes binding preferences to certain types of cells. Exosomes contain a variety repertoire of proteins, nucleic acids, making them multi-functional molecules. Created with https://biorender.com/.

Various methods have been developed to load diverse payloads into exosomes, including siRNAs, miRNAs, mRNA, circRNA, CRISPR/Cas9, proteins, and therapeutic drugs, using either passive or active loading^21,22^ (Figure 1). Relatively small molecules can be readily encapsulated into exosomes via the transient interruption of membrane integrity induced by various physicochemical methods. However, macromolecules with high molecular weights cannot be efficiently encapsulated using these passive methods. In this study, we specifically focus on strategies for loading protein therapeutics in their free forms to facilitate intracellular delivery.

### Active loading of free-form therapeutics for enhancing pharmacodynamic properties

**Figure 2.**
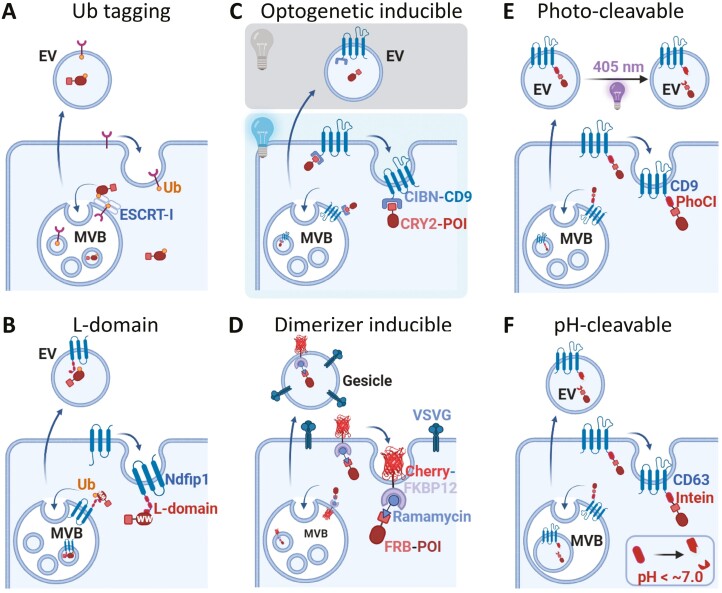
EV-loading strategies of free-cargo proteins. (A and B) involves the loading strategies mediated by ubiquitin-mediated interactions. (A) Ubiquitin tag, which is fused in the C-terminal tail of the cargo, is recognized by ESCRT complex for exosomal loading. (B) L-domain of an ILV marker protein interacts with a WW-domain in the cargo protein, facilitating mono ubiquitination of the cargo, followed by loading into intraluminal vesicles (ILVs). (C and D) utilizes light/chemical-induced protein–protein interaction (PPI). Photoactivated protein interactions occur between CRY-CIBN in the presence of light in (C) or FRB/FKBP in the presence of a rapamycin analog in (D). These reversible PPIs enable cargo loading into ILVs (one of PPI modules is connected to an ILV-enriched protein). Finally, (E and F) employ a protein-cleavable system. The cargo is covalently fused to the end of tetraspanin via photo- or pH-induced cleavage motif. In (E), the cargo is released from tetraspanin proteins by illumination of violet light to the purified EVs. In (F), the cargo is self-cleaved along with pH drop upon introduction into the ILVs. Created with https://biorender.com/.

A straightforward method for loading free-form proteins into exosomes is to directly transfect the genes of interest into exosome-producing cells. Normally, proteins overexpressed in cells are packed into exosomes via passive processes.^19^ However, owing to the relatively small volume ratio of exosomes (1.5 × 10^−15^ mL per particle) compared to producing cells,^23^ the efficiency of passive diffusion as a loading strategy is significantly limited. To address these challenges, several strategies have been developed for the active loading of free-form proteins into exosomes, based on ubiquitination, protein–protein interactions (PPIs), or protein-cleavable systems (Figure 2).^20,24-26^ One strategy involves the use of a ubiquitination-mediated protein-sorting system within exosome-producing cells (Figure 2A and B). Efficient transport of soluble proteins into the exosomal lumen has been achieved by fusing an engineered ubiquitin tag lacking the last 2 glycine residues in the C-terminus to the proteins of interest.^26^ Another approach utilizes the evolutionarily conserved late-domain (L-domain) pathway. In this system, a WW tag (W for tryptophan) is fused to the Causes Recombination (CRE) recombinase and recognized by the L-domain-containing protein Ndfip1, leading to its ubiquitination and subsequent loading into exosomes.^20^

The second strategy involves the induction of reversible PPI by chemical or optical means.^24,27,28^ This approach enables the loading of soluble protein cargo into exosomes through their interaction with membranes or membrane-associated proteins in exosomes, in the presence of specific chemicals or light (Figure 2C and D). One notable method within this category is exosome engineering for protein loading via optically reversible protein–protein interactions (EXPLOR), which utilizes the photoreceptor cryptochrome 2 (CRY2) and its interaction partner, CIBN.^24^ Here, cargo proteins are fused to CRY2, whereas the CIBN protein is fused to the exosomal membrane protein CD9, resulting in a reversible PPI upon blue light exposure. This in turn directs the CRY2-fused cargo protein to the inner surface of the exosomes via interactions with CIBN in the CIBN-CD9 complex.^24^ Another approach within this strategy involves FKBP-rapamycin binding domain (FRB) and FK506-binding protein (FKBP) complex formation mediated by a rapamycin analog.^28^ One example of this approach is demonstrated in a study using “Gesicles,” which are exosomes produced by the overexpression of viral spike glycoprotein vesicular stomatitis virus G (VSV-G). Ilahibaks et al successfully loaded cargo proteins into gesicles by fusing VSV-G to FKBP12 (DmrA) and fusing the T82L mutant FRB (DmrC) to the cargo.^27^ Notably, VSV-G, a viral fusogenic protein, aids the endosomal escape of these engineered gesicles within recipient cells, thereby enhancing the delivery efficacy of the therapeutic cargo.

A new strategy incorporating protein-cleavable systems has been proposed for active loading of therapeutic proteins. One approach utilizes a light-induced protein-cleavage system.^25,29^ By genetically fusing CD9 to a photocleavable protein (PhoCl) that splits into 2 fragments upon violet-light-induced cleavage, proteins, such as mCherry fluorescent protein, apoptin (an apoptosis-inducing protein), and catalase (an antioxidant enzyme), can be effectively loaded into exosomes, with the subsequent release of these proteins triggered by violet light^25^ (Figure 2E). Another method utilizes a self-cleaved mini-intein (intein) derived from *Mycobacterium tuberculosis* (Mtu) recA (Figure 2F). By genetically introducing inteins between exosomal membrane proteins, such as CD63 or VSV-G, and the desired cargo, efficient loading of cargo into exosomes is achieved, leading to successful delivery to target cells in vitro and in vivo.^30^

### Exosomal surface engineering for enhancing pharmacokinetic properties

To display targeting ligands, such as intrabodies, protein domains, or short peptide sequences, on the surface of exosomes, a common method involves incorporating these domains into exosome-targeting proteins. Examples of such targeting proteins include single transmembrane proteins (Lamp2b, VSV-G, PDGFRs, and PTGFRN), tetraspanins (CD63, CD9, and CD81), glycosylphosphatidylinositol-linked proteins, and phosphatidylserine (PS)-binding domains (lactadherin [C1C2 domain]).^31-35^ For example, a neuron-specific targeting peptide, rabies viral glycoprotein (RVG) peptide, was fused to the N-terminal region of Lamp2b, resulting in RVG on the exosomal surface. Subsequently, RVG-displaying exosomes derived from dendritic cells successfully delivered cargo to mouse neurons and brain.^31^ In another study, the PS-binding domains of lactadherin (C1C2) were fused to anti-epidermal growth factor receptor (EGFR) nanobodies to form an EGa1-C1C2 recombinant protein.^32^ These EGa1-C1C2 proteins, upon binding to the PS on the exosomal surface, showed enhanced specific binding and uptake by EGFR-overexpressing tumor cells in a dose-dependent manner.

Tetraspanins, such as CD9, CD63, CD81, and CD82, have 4 membrane-spanning domains, with both the N- and C-termini oriented toward the luminal side of the exosomes. The extracellular loops, namely, the short extracellular loop and large extracellular loop, are located on the outer surface of exosomes. Targeting moieties and peptides can be displayed on exosomal surfaces by incorporating targeting peptides into the extracellular loops of tetraspanins.^36^ For example, CD9-ApoB exosomes, in which ApoB-derived peptides are inserted between the 170 and 171 amino acids of CD9, demonstrate enhanced capability to target the central nervous system (CNS) in mice.^36^

Click chemistry, specifically copper-catalyzed azide-alkyne cycloaddition, is an efficient covalent reaction that forms a stable triazole linkage by combining an alkyne and azide residue.^37^ This technique can be used to attach different targeting moieties to exosome surfaces. In a mouse stroke model, exosomes labeled with the c(RGDyK) peptide through click chemistry exhibited an 11-fold increase in delivery to the ischemic region of the brain compared with exosomes labeled with scrambled peptides.^37^

## A lesson from preclinical studies of an anti-inflammatory exosome

The reticuloendothelial system (RES) is a network of innate immune cells such as macrophages and monocytes, located in liver, spleen, and lymph node. It recognizes foreign substances as well as nanoparticles and clear them via phagocytosis. Multiple studies have shown that intravenously injected exosomes are mostly taken up by macrophages via the negatively charged phosphatidylserine (PS) on the exosome surface.^38,39^ Therefore, inflammatory diseases are often selected as the initial indication for the application of engineered exosomes, by harnessing this natural tropism.

For example, CD24 glycoprotein, which inhibits NF-κB signal by the interaction with siglec-10 or damage-associated molecular patterns is displayed on the exosomes by overexpression to the producing cells (Exo-CD24). In its open-label phase I study (ClinicalTrials.gov identifier: NCT04747574), COVID-19 patients with mid/high severity administered with Exo-CD24 showed better recovery of the blood oxygen saturation rate with reduced blood inflammatory markers.^40^ There were no adverse events reported. Another example of anti-inflammatory exosomes is using decoy receptors for pro-inflammatory cytokines such as tumor necrosis factor receptor and/or Interleukin 6 cytokine family signal transducer (IL6ST). Those exosomes showed better clinical outcomes compared to preexisting TNF-α or IL6 biologic inhibitors in mouse models of sepsis, experimental autoimmune encephalomyelitis and inflammatory bowel disease.^41^

Our group has developed a novel anti-inflammatory exosome loaded with a dominant active form of endogenous nuclear factor-kappaB (NF-κB) inhibitor protein, IκB. This dominant active mutant of IκB, so-called super repressor IκB (srIκB), was developed in the late 1990s by replacing 2 serine residues responsible for signal-dependent ubiquitination and subsequent degradation with phosphorylation-resistant alanine residues.^42^ Owing to its superior selectivity compared to chemical inhibitors, srIκB has been widely used not only for in vitro experiments but also for in vivo models by genetic modification or adenoviral delivery. However, the absence of a safe and efficient delivery tool in humans significantly limits the potential use of this well-proven specific inhibitor of NF-κB in humans. We have shown that exosome-based delivery of srIκB can effectively suppress inflammatory responses induced by lipopolysaccharides (LPS) and tumor necrosis factor alpha (TNF-α) in vitro and ameliorate inflammation-related pathologies in various disease models, including sepsis, preterm birth, reperfusion-induced acute kidney injury, alcohol-induced liver damage, and chronic post-ischemia pain.^43-46^

Recently, our group has focused on the clinical translation of this novel anti-inflammatory exosome (Exo-srIκB, ILB-202). Using the EXPLOR technology, we genetically engineered Expi293F to produce Exo-srIκB. Large-scale production of GMP-grade exosomes was achieved using a combination of ultrafiltration and diafiltration, followed by anion exchange chromatography (AIEX) and size exclusion chromatography. The final products were subjected to comprehensive batch characterization, including tests for microbial contamination, endotoxin concentration, amount of cargo loaded per exosome, purity, morphology, and in vitro potency.^47^

The pharmacokinetic profiles and biodistribution patterns have been studied using exosomes conjugated with radioisotope Zr^89^. Biodistribution and plasma half-life were monitored in mice and rats by detecting radioisotope signals via whole-body positron emission tomography/computed tomography (PET/CT) or ex vivo gamma counting of selected organs.^47^ After a single intravenous administration of Zr^89^-labeled Exo-srIκB, the most pronounced signal was detected in the liver, followed by spleen, kidney, and heart, which is consistent with previous reports.^38,48,49^ The biodistribution in the lungs, stomach, and urinary bladder was minimal and transient. Notably, the biodistribution of exosomes can be affected by the pathological conditions of the host, as evidenced by the significant accumulation of lipophilic dye-labeled naïve exosomes in the lungs of septic mice.^50^

**Figure 3.**
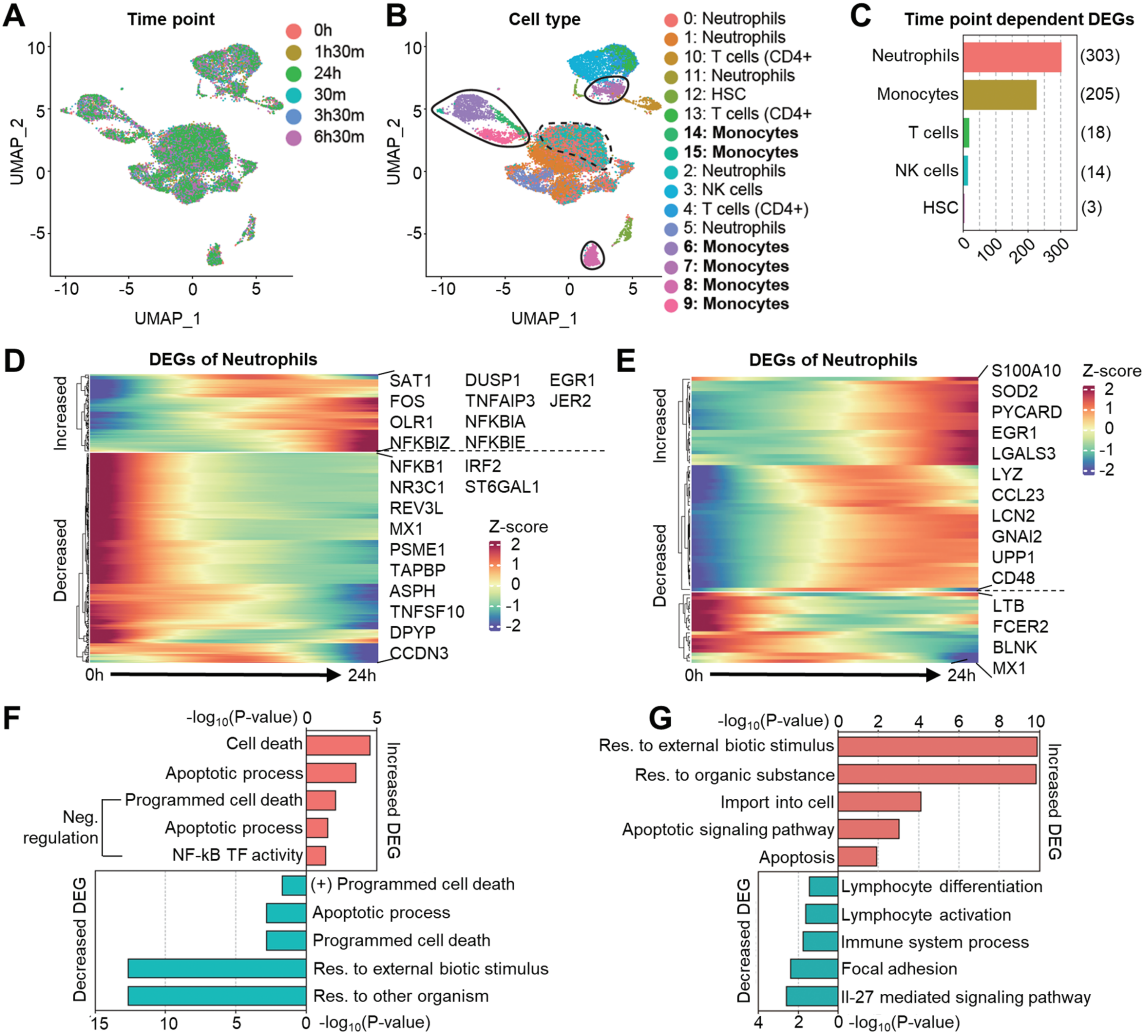
Beagle scRNA sequencing results show the prominent effect of exosomes in neutrophils and monocytes among blood cells. (A and B) UMAP embedding of analyzed transcriptomes of 17 915 white blood cells colored by (A) the time point at which each cell was prepared after exosome infusion, (B) cell cluster groups along similarities in transcripts expression, and the cell types annotated with reference to human primary cell atlas. (A) 3251, 2688, 3568, 3105, 2867, and 2906 cells were prepared at 0 hour, 30 minutes, 1.5 hours, 3.5 hours, 6.5 hours, and 24 hours after exosome infusion. (B) Areas with black lines, monocyte-specific regions; an area with black dashed lines, region of monocytes mixed with other cell types. (C) A graph indicating cell types with the number of DEGs. (D) Expression level heatmap for statistically significant NF-κB signaling related factors of neutrophils. (E) Expression level heatmap for statistically significant NF-κB signaling related factors of monocytes. (F) Gene ontology (GO)-biological process (BP) terms of DEGs with significant changes in expression levels shown in (D). (G) Gene ontology (GO)-biological process (BP) terms of DEGs with significant changes in expression levels shown in (E).

**Figure 4.**
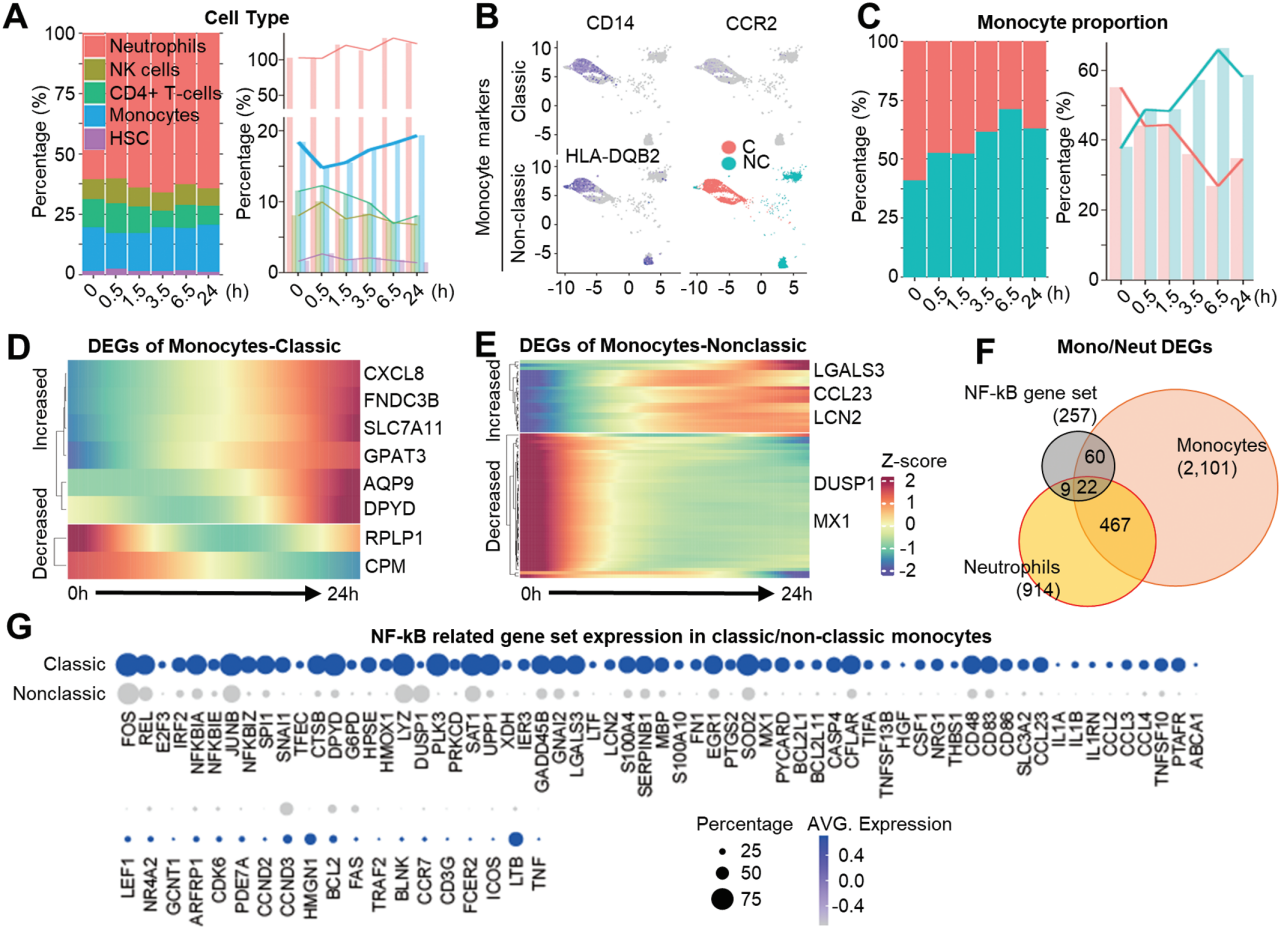
Beagle scRNA sequencing results show the non-classical monocytes prominently affected by the exosome. (A) A bar plot indicating cell type proportion. Hematopoietic stem cells (HSCs). (B) Monocyte clusters are further grouped with classic or non-classic monocyte marker genes. (C) A bar plot indicating cell type proportion. (D) Expression level heatmap for statistically significant NF-κB signaling related factors of classical monocytes. (E) Expression level heatmap for statistically significant NF-κB signaling related factors of non-classical monocytes. (F) Venn diagram showing DEGs in monocytes, neutrophils, or NF-κB gene set. The numbers in parentheses are the number of DEGs from each population. (G) The proportions and expression patterns of 82 differentially expressed NF-κB genes in classical or non-classical monocytes are depicted as the size and color of the circles, respectively.

With a favorable preclinical toxicity profile, ILB-202 was recently approved for use in a single-center, randomized, double-blind, placebo-controlled clinical trial as a single ascending dose for healthy participants in Australia (ClinicalTrials.gov identifier: NCT05843799). To the best of our knowledge, this is the first clinical trial using engineered exosomes for systemic intravenous administration. While the main purpose of this phase I clinical trial was to evaluate the safety and tolerability, efforts have also been made to identify biomarkers that can represent the pharmacodynamic properties of ILB-202, even in healthy volunteers. To this end, we analyzed whether a single injection of ILB-202 could change the expression profiles of NF-κB-related genes in the leukocytes of healthy animals. This approach was conceived based on the finding that the mononuclear cells and neutrophils were the main cell types responsible for the anti-inflammatory effect induced by Exo-srIκB in our preclinical studies.^43,44^ Beagle dogs were administered a therapeutic dose (9.3 × 10^10^ pn/kg) of Exo-srIκB via intravenous infusion for 30 min, and peripheral blood was drawn at several time points (0 h, 0.5 h, 1.5 h, 3.5 h, 6.5 h, and 24 h) pre- and post-infusion for leukocytes isolation and subsequent transcriptome analysis via single-cell RNA sequencing (scRNA-seq) (Figure 3, unpublished data). After the enrichment of white blood cells by red blood cell lysis, we analyzed a total of 17 915 cells (Figure 3A). Uniform manifold approximation and projection analysis followed by unsupervised clustering revealed the 15 cell populations (Figure 3B). To examine the effect of ILB-202 treatment on the expression patterns of various immune cells, we first evaluated the number of significantly differentially expressed genes (DEGs) and found that neutrophils and monocytes showed the highest number of DEGs after ILB-202 treatment (Figure 3C). Among those genes, there was a noticeable down-regulation of a subset of NF-κB-related genes during the early time points in both neutrophil and monocyte clusters (Figure 3D, E, G, and H). There was also a subset of genes that increased upon ILB-202 treatment. Given that the autoregulatory feedback loops for NF-κB are tightly controlled by the activity of NF-κB,^51^ the increase in the expression of NF-κB-related genes may suggest reduced activity within the NF-κB signaling pathways. After the administration of ILB-202, there was a continuous increase in the total monocyte population after a transient decrease at 0.5 hours (Figure 4A). We further grouped a monocyte cluster into classical and non-classical monocytes based on their expression of established marker genes (Figure 4B). Strikingly, the non-classical monocytes, which are characterized by their anti-inflammatory behavior,^52,53^ increased in proportion after exosome injection, while the proportion of classical monocytes decreased (Figure 4C). Unlike the neutrophil subsets, the numbers of DEGs of classical or non-classical monocytes were significantly lower than that of DEGs of total monocytes (see Figure 3C and G). As expected, the expression level of 82 NF-κB-related genes was higher in the classical monocytes compared to the non-classical monocytes (Figure 4G). These results suggest that scRNA-seq analysis might be used to identify the major cell types responsible for exosome treatment and demonstrate the target engagement of Exo-srIκB, which is the NF-κB signaling in the mononuclear cells.

Taken together, we demonstrate the potential of the scRNA-seq technique in preliminary pharmacodynamic investigations, even among healthy individuals. Currently, clinical studies on ILB-202 are underway in healthy human participants, and PBMCs have been collected for scRNA-seq analysis. We hope to report phase I clinical trial results along with a pharmacodynamic study in the near future.

## Therapeutic application of engineered exosomes

### Therapeutic application for CNS diseases

As mentioned above, exosomes preferentially target innate immune cells, such as monocytes or neutrophils. However, active targeting is required when the indicated diseases are not limited to immune cells, such as in neurodegenerative disorders. This was evident from the aforementioned in vivo biodistribution experiments using radioisotope Zr^89^.^47^ When mice and rats were monitored after a single intravenous administration of Zr^89^- Exo-srIκB, only less than 0.1% of Zr^89^-Exo-srIκB ended up reaching the brain, testes, and ovaries.^47^ The negligible distribution of exosomes in the brain is mainly due to the BBB, which is a unique structure consisting of tightly sealed endothelial cells covered by pericytes and astrocytes.^54^ Owing to the unusual tight junctions in endothelial cells, together with the multiple layers wrapping the vessels, drug delivery across the BBB is largely limited. Hence, active targeting via surface engineering is required for exosomal delivery across the BBB.

Currently, the most common strategy is to add targeting moieties (ie, antibodies, scFv, or peptides) to receptor proteins that are highly expressed in brain endothelial cells, such as the transferrin receptor, low-density lipoprotein receptor (LDLR), or LRP (LDLR-related protein). Thus, exosomes can cross the BBB via receptor-mediated transcytosis and deliver therapeutic cargo to targeted neurons.^55-57^ Light, particularly near-infrared (NIR) light, has long been studied as a stimulant for increasing BBB permeability. Recently, it was suggested that transcranial laser excitation along with gold nanoparticles could transiently open the BBB, allowing macromolecules to move across it.^58^ The reversible opening of the BBB, induced by light stimulation, has the advantage of modulating permeability in a spatiotemporal manner. Therefore, the application of surface-engineered exosomes under light stimulation at the right time and place is expected to synergistically enhance the brain delivery of exosomes and would be helpful in treating neurodegenerative CNS diseases, such as Alzheimer’s disease, tauopathy, and multiple sclerosis (MS).

Alzheimer’s disease is the most common cause of dementia and is characterized by the accumulation of amyloid-beta (Aβ) plaques and hyperphosphorylated tau proteins in the brain.^59^ Aβ plaques are precipitated in the extracellular space and form insoluble aggregates that disrupt neuronal function.^60,61^ Tau pathology involves abnormal phosphorylation of the tau protein, leading to its aggregation into neurofibrillary tangles. Abnormal tau seed fragments can be transported to the neighboring neurons in the form of exosomes.^62,63^ The continuous failure of approaches to target Aβ plaques has raised doubt on the so-called amyloid-beta hypothesis.^64,65^ Although the recently FDA-approved monoclonal anti-Aβ antibodies lecanemab and aducanumab have provided a silver-lining, they are only recommended for early-stage patients to slow the disease progression. To date, there are no FDA-approved drugs that target tau. Targeting tau with the current approach is even more difficult than targeting Aβ because neurofibrillary tangles of tau accumulate inside the cells, which antibodies cannot penetrate. Therefore, antibody-loaded exosomes are an attractive alternative delivery system.

Multiple sclerosis (MS) is a chronic auto-immune disease that affects the central nervous system, where myelin, the protective layer of the nerve fibers, is damaged by the attack from own immune system. This can lead to a range of symptoms, including problems with motor function, sensation, vision, and balance.^66,67^ Innate immune cells as well as adaptive immune cells (eg, T cells) contribute to the progression of MS.^68,69^ Approved drugs are mostly disease-modifying treatments with low efficacy and frequent side-effects. Since exosomes have natural tropism to innate immune cells, infusion of anti-inflammatory exosomes will target activated microglia and astrocytes in the CNS and might reduce the severity of MS pathology by reducing inflammation.

Recently, there have been significant advances in targeted protein degradation (TPD), which degrades proteins of interest via the ubiquitin-proteasome pathway (PROTACs), endolysosomal pathway (LYTACs), and autophagosome-mediated degradation (AUTACs or AUTOTACs).^2^ To degrade aggregated proteins, such as Aβ or neurofibrillary tangles, lysosomal or autophagosome-mediated TPD would be much more appropriate. LYTAC can be applied to Aβ removal as it is specialized in bringing extracellular or membrane-bound proteins into the lysosome, while AUTAC or AUTOTAC can be applied to degrade intracellular neurofibrillary tangles of tau. With these heterobifunctional degraders, we can expect treatment to not only prevent aggregate formation but also remove preexisting aggregates, making TPD technology a better option for treating Alzheimer’s disease. In case of MS, therapeutic exosomes loaded with PROTACs against JAK/STAT signaling (eg, STAT3) would be beneficial since it can induce targeted degradation of STAT3 in microglia- or astrocytes-specific manner, improving the efficacy of drugs while reducing side-effects.^70^

Most developed heterobifunctional degraders are chemical-based, consisting of a target-binding warhead, target-recruiting ligand, and linker. One of the disadvantages of chemical-based heterobifunctional degraders is their selectivity and off-target effects.^71,72^ To ameliorate these side effects and increase on-target specificity, conversion to biologics is achieved by constructing the target recognition domain with antibodies, scFv, or VHH, and a proteolysis-inducing domain with E3 ligases or E3-ligase-binding peptides fused with linkers in between.^73,74^ The delivery problem of biologic heterobifunctional degraders can be solved by loading them into exosomes; therefore, surface-engineered exosomes loaded with biologic heterobifunctional degraders will open a new era of exosome-based therapeutics for TPD.

### Therapeutic application for cancers

The development of exosome therapeutics for cancers needs careful consideration of diverse issues including tumor-specific targeting without non-specific uptake in the liver, administration route, and number of doses for different types of cancer. Since this is a huge topic and beyond the scope of this review, we will cover topics related to tumor-associated inflammation. While many anticancer drugs can block hyperactivated signaling pathways (ie, AKT, JAK/STAT, WNT, and Hippo signaling pathways) in tumor cells, including chemical inhibitors or antisense oligonucleotides (ASO), these pathways are also important in maintaining normal homeostasis. These findings support the importance of targeted drug delivery. While antibody drug conjugates confer specificity to a particular target, toxicity issues remain due to deconjugation and unwanted payload release after administration.^75^ In contrast, surface-engineered exosomes demonstrate improved tumor targeting by displaying tumor antigen-binding moieties on their surface.^33,76-78^ Additionally, when combined with pH-sensitive fusogens, they may enable cancer-specific cargo release.^79^ Because the tumor microenvironment is acidic, it may be possible to deliver exosomal therapeutics to cells that constitute the tumor microenvironment, such as tumor-associated macrophages and stromal cells. For example, delivery of biological heterobifunctional degraders for STAT3 can shift the tumor microenvironment toward an anti-tumor state, either by redirecting tumor-associated macrophages toward the inflammatory M1 type macrophages or by inducing stromal cells to produce inflammatory cytokines.^80^ This could also synergize with immune checkpoint therapies, such as anti-PD-1 or anti-PD-L1.

## Conclusion

Exosomes have strong potential as DDSs, albeit with the challenges mentioned above. Continued efforts to characterize exosomes and their subtypes using advanced techniques are required to demonstrate exosome delivery of drugs as successful therapeutics. In this review, we summarized (1) various exosome engineering strategies for protein cargo loading and active targeting, (2) progress toward the clinical transition of engineered exosomes as anti-inflammatory therapeutics, and (3) the potential therapeutic applications of exosomes in treating other diseases, such as CNS diseases and cancer.

Notably, exosomes are highly heterogeneous; thus, the proteins abundant in exosomes may vary depending on the cell lines and purification methods. Exosome production efficiency and target tropism also vary among cell lines. Therefore, for consistency in the therapeutic effects of exosomes, careful consideration should be paid to the selection of cell lines for exosome production and purification methods prior to mass production. Pharmacokinetic studies that monitor the biodistribution of exosomes are critical for determining disease indications. Monitoring biodistribution using lipophilic dyes or radiolabeled exosomes does not specify the major cell types targeted within the organs or tissues of interest. In this context, scRNA-seq enables the observation of the cellular dynamics of all cell types within a specific organ or tissue upon exosome treatment. Moreover, changes in gene expression in specific cell types can be easily analyzed under a variety of conditions.

There are many efforts to develop engineered exosomes as therapeutics and a couple of pipelines are in clinical trials, confirming their tolerability and safety. So far, most of the preclinical and clinical pipelines are related to inflammatory diseases because exosomes can target innate immune cells naturally even without additional surface modification. To expand the disease spectrum beyond inflammation, tissue- or cell-type-specific targeting moieties must be introduced along with nonspecific phagocytosis-avoidance strategies. The introduction of biological degraders as exosome payloads might provide valuable treatment options to solve proteopathy-associated degeneration. In the near future, next-generation exosome therapeutics are expected to overcome the limitations of conventional therapies for cancer or degenerative diseases.

## Data Availability

All data included in this study are available upon reasonable request from the corresponding authors.
